# Food and Age: It Takes Two to Degenerate

**DOI:** 10.3389/fnagi.2020.00182

**Published:** 2020-06-26

**Authors:** Raneen Nicola, Eitan Okun

**Affiliations:** ^1^The Leslie and Susan Gonda Multidisciplinary Brain Research Center, Bar-Ilan University, Ramat Gan, Israel; ^2^The Paul Feder Laboratory on Alzheimer's Disease Research, Bar-Ilan University, Ramat Gan, Israel; ^3^The Mina and Everard Goodman Faculty of Life Sciences, Bar-Ilan University, Ramat Gan, Israel

**Keywords:** aging, intermittent fasting, neurodegeneration, autophagy, Alzheimer's disease, caloric restriction

## A Blessing and a Curse

**Figure 1:**
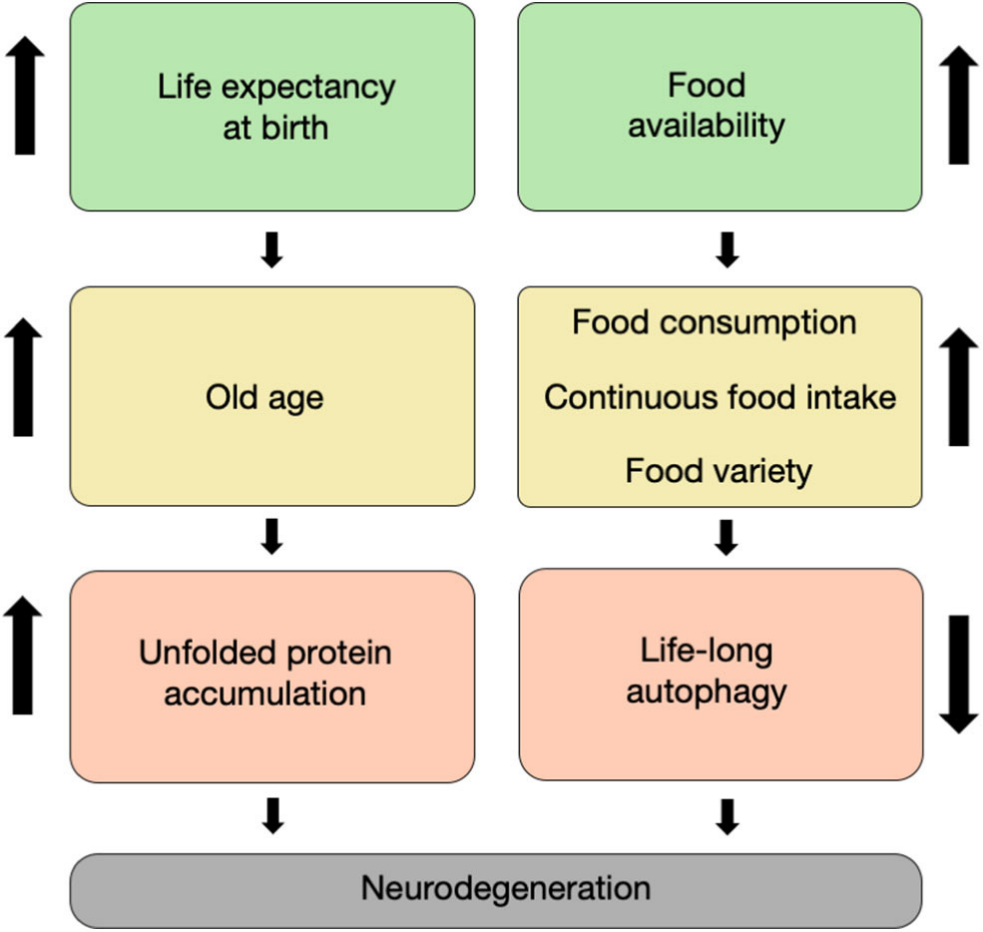
It takes age and food to degenerate. The increased life expectancy at birth witnessed throughout the last two centuries has led to an increase in the proportion of elderly in the population, which is characterized by unfolded proteins and non-functional organelle accumulation within neurons (left column). Concomitantly, the increase in food availability during the second half of the twentieth century led to an increase in food varieties, consumption, and intake. This has led in turn to a decrease in life-long autophagy flux in cells, including neurons (right column). Both of these routes have synergized to result in a large increase in the prevalence of neurodegenerative disorders that we currently witness.

The twentieth century has brought numerous advances in technology, medicine, and food security. The reduced mortality of infants, toddlers, adults, and the elderly due to technological breakthroughs in medicine has brought a stable increase in the global expected lifespan at birth (Figure 1). Specifically, worldwide, the life expectancy of males rose from 59.6 years in the 1980's to 69.0 years in 2015, whereas the life expectancy of females increased from 63.7 to 74.8 years, respectively (Mortality and Causes of Death, 2016). This increase in lifespan is correlated with multiple age-dependent pathologies which have also increased in prevalence, such as neurodegenerative disorders (Hebert et al., 2013).

While food fermentation as a means to attain food security occurred as early as 5,000 years ago, fermentation processes are believed to have been developed in order to preserve fruits and vegetables for times of scarcity in which food availability was intermittent (Medina-Pradas et al., 2017). This is not the situation nowadays, as food fermentation is not required for securing food anymore, but rather supplementing already available food stocks. The global increase in food security due to modern long-term food storage coupled with the increase in worldwide global food transportation, and international marketing has reduced the cost of food, increasing its availability in the developed world (Barnard, 2010) (Figure 1). However, food commercialization and the shift toward production of processed and ultra-processed foods have revealed clear adverse effects, such as the identification of processed food as a major cause for over-eating and the increase in the risk of metabolic syndrome, obesity, and diabetes (Hall et al., 2019). As the brain is one of the primary energy-demanding organs in the human body, it comes with no surprise that the brain is highly affected by such metabolic disorders as evident by recent epidemiological studies (Beydoun et al., 2008; Mule and Singh, 2018). For example, type-2 diabetes is strongly associated with cognitive impairment due to insulin resistance and altered glucose availability to neurons, which impair energy production capacity and proper neuronal function (Kandimalla et al., 2017).

Therefore, it's plausible to argue that the combined effect of the continued increase in lifespan and life-long continuous food consumption leads to a dramatic increase in the prevalence of neurodegenerative disorders in the elderly population. Herein, we will discuss factors that have shifted our nutritional habits over the last century. Next, we will delineate the effects of nutritional imbalance on neurodegenerative diseases at the cellular level, by shedding a light on the autophagy regulation. Moreover, we will discuss how Alzheimer's disease (AD), the most prevalent neurodegenerative disorder, estimated to affect 55 million Americans aged 65 and above, can be affected by such changes (Hebert et al., 2013; Brookmeyer et al., 2018).

## A Century of Medical Revolutions

Early in the twentieth century, life expectancy at birth in most developed countries ranged between 45 and 50 years, with significant numbers of young children not reaching the age of 10, mostly due to infectious diseases. Throughout the course of the last 60 years, however, the average lifespan at birth has risen linearly (Bell and Miller, 2005; Dong et al., 2016). Many factors can account for this, including early developments such as sanitation and clean water, which dramatically decreased infant mortality. Additional reasons include vaccine development against infectious agents, which significantly decreased children mortality from infections, the development of therapies for cardiovascular complications and cancer in older adults, and finally, the development of life-supporting devices for frail elderly people. As a result, over 95% of infants born in developed countries today will live to age 50 years or older, and over 84% of them will survive to age 65 years or older (Bell and Miller, 2005; Olshansky, 2018).

Although the maximal lifespan of humans has been shown by some mathematical models to be fixed (Dong et al., 2016), maximal lifespan at birth is still rising linearly, as for 160 years, the maximal life expectancy has witnessed consecutive increases by a quarter of a year per year (Oeppen and Vaupel, 2002).

## Globalization and Food Security

One of the outcomes of the industrial, technological and social changes in the twentieth century is the steep increase in the availability of food products, many of which are ready-made, and the consumption of foods of low-nutritional values and processed foods (Figure 1). Furthermore, it has been recently shown, in a well-controlled study in humans, that consumption of ultra-processed food [according to the NOVA system of food classification (Monteiro et al., 2018)] leads to increased caloric intake (Hall et al., 2019), which is thought to be at the basis of the obesity epidemic in the western world.

Children's food and beverage preferences and consumption are largely affected by media advertisements. Indeed, acute exposure to food advertising increases food intake specifically in children rather than in adults (Boyland et al., 2016), thus increasing the proportion of obese children who are at greater risk to develop type-2 diabetes at adulthood (Kelsey et al., 2014), which in itself is proposed to correlate with dementia (Biessels and Despa, 2018).

## Eating Ourselves Away

Aging is accompanied by a deterioration in multiple physiological aspects and can be characterized by cellular and molecular hallmarks, such as genomic instability, loss of proteostasis, cellular senescence, and more (López-Otín et al., 2013). This age-dependent decline in functioning also occurs in the brain, which becomes more vulnerable to oxidative stress, inflammatory insults, and metabolic stress. Moreover, multiple processes such as protein folding, degradation, and nutrient-sensing may be damaged (Kemnitz et al., 1994; López-Otín et al., 2013; Cenini et al., 2019). This age-dependent decline in functioning is a major risk factor for several neurodegenerative diseases such as AD, the most prevalent neurodegenerative disorder (Oddo, 2012; Cenini et al., 2019) (Figure 1). Another possible risk factor of neurodegenerative diseases is high caloric intake and obesity (Luchsinger et al., 2002; Beydoun et al., 2008). Accumulating evidence indicates that excessive food consumption may be harmful to the brain as continuous high glucose levels may increase oxidative stress, harming the vulnerable aging brain (Mule and Singh, 2018).

Studies in laboratory animals show that caloric restriction (decreased food intake or intermittent fasting) can extend lifespan in rodents and primates (Anderson et al., 2009; Colman et al., 2014) and delay the onset of age-related diseases such as hypertension and diabetes (Anderson et al., 2009; Colman et al., 2009; Fontana et al., 2010). Moreover, caloric restriction may protect neurons from degeneration and enhance adult neurogenesis and neuronal plasticity, which may protect the brain from a cognitive decline during aging and neurodegenerative diseases (Duan et al., 2001; Mattson et al., 2003) (Figure 1). It is still uncertain, however, how forms of caloric restriction or intermittent fasting affect Amyloid-beta (Aβ) oligomerization and deposition and behavioral deficits in various AD rodent models. For example, both short- and long-term caloric restriction in APP/PS1 mice significantly decreased the accumulation of Aβ (Patel et al., 2005; Mouton et al., 2009), while opposite data were shown when similar interventions were tested in the 5XFAD mouse model of AD (Lazic et al., 2020). Similar beneficial effects of caloric restriction were shown in animal models of Parkinson's Disease (PD) (Duan and Mattson, 1999; Maswood et al., 2004), providing evidence for a generalized role of reduced caloric intake in ameliorating neurodegeneration.

One of the crucial processes that are adversely affected during aging is cellular autophagy (Anderson et al., 2009; Rubinsztein et al., 2011), which is tasked with eliminating aggregated proteins, unhealthy organelles, and multiple intracellular components through their isolation in autophagosomes and fusion with lysosomes for breaking down these components (He and Klionsky, 2009; Rubinsztein et al., 2011). This process allows the recycling of cellular macromolecules, which can be used to maintain energy and proper cell functioning (Kim and Guan, 2015). Accumulation of autophagic vesicles, which may contain undigested misfolded proteins, characterizes many neurodegenerative diseases, such as PD and AD (Lee et al., 2011).

Multiple mechanisms can explain the roles of fasting and caloric restriction in ameliorating neurodegeneration. One of the most studied mechanisms is the upregulation of autophagy via inhibiting mTOR activity, which promotes anabolic metabolism, necessary for protein synthesis and proliferation and cell growth, and inhibits catabolic activity (Kim and Guan, 2015). The mTOR pathway is activated by nutrient cues, such as amino acids, glucose (Gonzalez and Hall, 2017), and fat (Menon et al., 2017), which are abundant following food intake. Signaling events downstream to mTOR are involved in inhibition of cellular autophagy, a process that eliminates unfolded proteins and organelles within cells (Kim and Guan, 2015). This effect on mTOR activity could be mediated through two important energy sensors, namely, AMP-activated protein kinase (AMPK) and Sirtuin-1, and/or through decreasing downstream signaling by the insulin growth factor (IGF)-receptor (Rubinsztein et al., 2011). Thus, constant uptake of nutrients results in continuous activation of the mTOR pathway while concomitantly transcriptionally inhibiting the autophagy pathway, leading to life-long accumulation of unfolded proteins, a process that could promote neurodegeneration. These insights suggest that life-long enhancement of autophagy, whether by dietary or pharmacological means, can potentially prove vital to delaying the onset of neurodegenerative disorders in the elderly population.

While intermittent fasting and caloric restriction emphasize the effect of the timing of food consumption and food quantity on health and disease, other interventions, such as the ketogenic diet, focus instead on food quality. The concept of food quality vs. quantity in ketogenic diet cannot be dissociated, as ketogenic diet was shown to stimulate autophagy in the CNS (Mcdaniel et al., 2011) due to reduced circulating glucose and insulin levels (Paoli et al., 2014) while reducing mTOR activity (Mcdaniel et al., 2011). In the case of ketogenic diet, the elevated ketone bodies in the circulation are used as an alternative energetic metabolite to the brain, which is thought to be responsible for the beneficial effects of ketogenic diet in neurodegenerative disease such as AD (Kashiwaya et al., 2013; Wlodarek, 2019).

In the past decades, significant knowledge has accumulated regarding the importance of quantitative nutritional limitation of food consumption on maintaining proper homeostatic metabolism pathways, namely mTOR and autophagy, in various cell types and tissues. There is still a lack of deep understanding of the mechanisms regulated in the metabolically unique cellular environment of the brain, chiefly neurons, and astrocytes. Furthermore, although numerous studies have associated the consumption of certain types of nutrients with shortening or expanding lifespans in animals including primates (Mattison et al., 2017; Di Francesco et al., 2018; Wahl et al., 2018), the neurobiology community should move forward into studying how this applies to brain maintenance and function in young vs. aged animals. Moreover, causal, and not only associative links, between different food types and metabolic pathways in the brain should be studied. Lastly, we hope that new avenues of research into the impact of nutritional quantities and types on the aging human brain will be studied more rigorously in the near future, in order to provide the community with better tools for managing or even delaying the increasing rates of neurodegenerative diseases.

## Concluding Remarks and Future Perspectives

The sobering statistics of one in three elderly people suffering from a type of age-related dementia call to devise a multi-pronged approach to targeting age-related neurodegenerative diseases. Synthesis of the current data indicates that not only age but also dietary lifestyles that changed dramatically during the twentieth century are at play. An expanding body of literature correlates dietary interventions with longevity. Indeed, many factors that are at play during aging have a role in promoting neurodegeneration, such as oxidative stress, accumulation of DNA damage, cell senescence, neuro-inflammation, and decreased autophagic flux. Furthermore, most of these factors have both intrinsic and extrinsic drivers behind them. For example, aging, characterized by impaired sleep patterns (Mander et al., 2017), has been shown to mediate impaired DNA repair (Zada et al., 2019). Aging is also characterized by elevated levels of neuroinflammation that are transcriptionally regulated (Baruch et al., 2013). Autophagy, however, is a cellular pathway that throughout life is predominantly regulated extrinsically in a nutrient-consumption mediated manner. This places food consumption as a major factor, along with aging itself, in promoting neurodegenerative disorders. As one of the main aims of dietary regimes, such as intermittent fasting, is to inhibit mTOR and promote autophagy, it is yet unknown what the optimal timing is for this intervention in relation to the circadian rhythm. Furthermore, it is plausible that future research into mTOR inhibition by Rapamycin analogs, for example, can efficiently replace dietary interventions.

## Author Contributions

EO and RN wrote the paper. All authors contributed to the article and approved the submitted version.

## Conflict of Interest

The authors declare that the research was conducted in the absence of any commercial or financial relationships that could be construed as a potential conflict of interest.
